# Effects of age and smoking on endothelial function assessed by quantitative cardiovascular magnetic resonance in the peripheral and central vasculature

**DOI:** 10.1186/s12968-015-0110-8

**Published:** 2015-02-19

**Authors:** Michael C Langham, Yongxia Zhou, Erica N Chirico, Jeremy F Magland, Chandra M Sehgal, Erin K Englund, Emile R Mohler, Wensheng Guo, Suliman Barhoum, Felix W Wehrli

**Affiliations:** Department of Radiology, University of Pennsylvania Medical Center, 3400 Spruce Street, Philadelphia, PA 19104 USA; Department of Medicine, University of Pennsylvania Medical Center, Philadelphia, PA USA; Department of Biostatistics and Epidemiology, University of Pennsylvania Medical Center, Philadelphia, PA USA

**Keywords:** Vascular reactivity, Oximetry, Velocimetry, Pulse wave velocity, Smoking, Aging, Cardiovascular magnetic resonance

## Abstract

**Background:**

Both age and smoking promote endothelial dysfunction and impair vascular reactivity. Here, we tested this hypothesis by quantifying new cardiovascular magnetic resonance (CMR)-based biomarkers in smokers and nonsmokers.

**Methods:**

Study population: young non-smokers (YNS: N = 45, mean age = 30.2 ± 0.7 years), young smokers (YS: N = 39 mean age 32.1 ± 0.7 years), older non-smokers (ONS: N = 45, mean age = 57.8 ± 0.6 years), and older smokers (OS: N = 40, mean age = 56.3 ± 0.6 years), all without overt cardiovascular disease. Vascular reactivity was evaluated following cuff-induced hyperemia via time-resolved blood flow velocity and oxygenation (SvO_2_) in the femoral artery and vein, respectively. SvO_2_ dynamics yielded washout time (time to minimum SvO_2_), resaturation rate (upslope) and maximum change from baseline (overshoot). Arterial parameters included pulse ratio (PR), hyperemic index (HI) and duration of hyperemia (T_FF_). Pulse-wave velocity (PWV) was assessed in aortic arch, thoracoabdominal aorta and iliofemoral arteries. Ultrasound-based carotid intimal-medial thickness (IMT) and brachial flow-mediated dilation were measured for comparison.

**Results:**

Age and smoking status were independent for all parameters. Smokers had reduced upslope (−28.4%, P < 0.001), increased washout time (+15.3%, P < 0.01), and reduced HI (−19.5%, P < 0.01). Among non-smokers, older subjects had lower upslope (−22.7%, P < 0.01) and overshoot (−29.4%, P < 0.01), elevated baseline pulse ratio (+14.9%, P < 0.01), central and peripheral PWV (all P < 0.05). Relative to YNS, YS had lower upslope (−23.6%, P < 0.01) and longer washout time (13.5%, P < 0.05). Relative to ONS, OS had lower upslope (−33.0%, P < 0.01). IMT was greater in ONS than in YNS (+45.6%, P < 0.001), and also in YS compared to YNS (+14.7%, P < 0.05).

**Conclusions:**

Results suggest CMR biomarkers of endothelial function to be sensitive to age and smoking independent of each other.

## Background

Cigarette smoking is a major modifiable risk factor for systemic atherosclerosis and thus cardiovascular disease (CVD) [1]. It is estimated that cigarette smoking is responsible for over 10% of CVD related deaths [2]. According to World Health Organization statistics, smoking was “the primary causal factor for at least 30% of all cancer deaths, for nearly 80% of deaths from chronic obstructive pulmonary disease, and for early cardiovascular disease and deaths during the period from 2000 to 2004” [3]. Based on Framingham heart study, the hazard ratio of smoking ranked second, next to diabetes, among all CVD risk factors [4].

Endothelial dysfunction (EDF), characterized by a reduction in the bioavailability of nitric oxide (NO), is the earliest stage of atherogenesis [5], resulting in increased inflammation, inadequate vasodilation, and thrombosis [6,7]. Various conditions, including hypertension, hypercholesterolemia, diabetes mellitus, etc., but also advanced age, result in reduced release of NO either because of impaired synthesis or bioavailability [8]. Cigarette smoking has long been known to produce reactive oxygen species (ROS) [9], which damage the endothelium, thereby causing a reduction in NO bioavailability and, in turn, impaired endothelium-dependent vasodilation [10].

Several non-invasive imaging methods exist for assessing the vascular manifestations of endothelial dysfunction. Flow-mediated dilation (FMD) measured by B-mode ultrasound in the brachial artery in response to cuff-induced ischemia is considered a surrogate measure of EDF [11], used in the research setting since the 1990s. Celermajer et al. found reduced FMD to be independently associated with cigarette smoking as was older age (P < 0.01) [12]. However, FMD is difficult to measure given the small changes in vessel diameter caused during hyperemia (typically 3-8%) [13] and intra-subject reproducibility is still a major challenge [14]. Further, changes in intima-media thickness (IMT) of the carotid artery measured via B-mode ultrasound, have provided important insight into early manifestations of atherogenesis [15]. IMT data show strong associations with cigarette smoking indicating increased values in active smokers, and to a lesser extent in past smokers and even in passive smoking [16]. However, there is also evidence that the method has limitations in terms of reproducibility, resulting from high inter- and intra- observer variability [15].

Another image-based metric of vascular health is pulse-wave velocity (PWV), a parameter that measures arterial stiffness [17,18], i.e. the propagation speed of the systolic pulse-wave measured from the delayed arrival of the pulse pressure wave at some downstream location. There are a number of studies comparing PWV or its surrogates between smokers and nonsmokers, with often conflicting results (see, Doonan et al. for a literature review [19]). The major limitation of conventional tonometry is that the path length of the pressure wave cannot be determined accurately. A small cardiovascular magnetic resonance (CMR) study compared distensibility, expressed as the change in cross-sectional area divided by pulse pressure in the aorta and common carotid artery, showing a significant reduction in young smokers relative to their non-smoking peers [20].

In this study, we applied new CMR methods developed in the authors’ laboratory that provide surrogate measures of vascular reactivity and endothelial dysfunction to evaluate effects of age and smoking on vascular health in subjects without symptomatic cardiac disease. We hypothesized that parameters characterizing micro- and macro-vascular reactivity are impaired in smokers relative to age-matched non-smokers in a manner similar to the effects that advanced age is expected to have on the vascular system. Methods incorporated into a single examination protocol include dynamic venous oximetry that makes use of venous oxygen saturation as an endogenous tracer [21] monitored during hyperemia, along with arterial velocity [22]. PWV is measured in the central and peripheral arteries using new projection imaging techniques [23,24]. Lastly, the CMR measures are compared with conventional ultrasound measures that include carotid IMT and brachial FMD.

## Methods

### Subjects

A total of 169 subjects divided into four groups were studied: young healthy non-smokers (YNS: N = 45, mean age = 30.2 ± 0.7 years, age range: 26–40 years), age-matched young smokers (YS: N = 39, mean age = 32.1 ± 0.7), older healthy non-smokers (ONS: N = 45, mean age = 57.8 ± 0.6 years, age range: 51–65 years), and age-matched older smokers (OS: N = 40, age = 56.3 ± 0.6). Full details of the study population are given in Table 1. All subjects had their resting heart rate, systolic and diastolic blood pressure measured upon enrollment and a lipid profile was obtained, and C-reactive protein assayed. All study participants were free of symptomatic cardiovascular disease. Subjects were requested to refrain from eating, exercise, taking vasoactive medications (including over-the-counter medications such as vitamin C), and smokers from smoking for 12 hours prior to the imaging procedures. Written informed consent was obtained prior to all examinations following an institutional review board-approved protocol. Total smoking exposure in terms of cigarette pack-years was 7.34 ± 0.76 and 12.57 ± 2.20 for young and older smokers, respectively.

**Table 1:** **Subject and cardiovascular characteristics (means ± standard error)**

**Groups**	**Y-NS (N = 45)**	**Y-S (N = 39)**	**O-NS (N = 45)**	**O-S (N = 40)**
**Age (years)**	30.2 ± 0.7	32.1 ± 0.7	57.8 ± 0.6	56.3 ± 0.6
**Men**	17	20	13	22
**White**	29	16	29	14
**Black**	6	21	9	26
**Asian**	10	2	7	0
**SBP (mmHg)**	115.4 ± 1.9**	116.7 ± 2.2	122.7 ± 2.7**	127.3 ± 2.2
**DBP (mmHg)**	73.4 ± 1.2	75.3 ± 1.9	76.7 ± 1.5	81.0 ± 1.5
**MAP (mmHg)**	87.5 ± 1.2	89.1 ± 1.9	91.6 ± 1.8	96.4 ± 1.6
**HR (bpm)**	75.6 ± 1.8	75.2 ± 2.3	75.9 ± 1.3	76.9 ± 1.7
**BMI (kg/m** ^**2**^ **)**	23.4 ± 0.5* **	26.1 ± 0.7*	26.2 ± 0.6**	26.5 ± 0.6
**Total Chol (mg/dL)**	170.5 ± 4.8**	172.7 ± 6.4	196.2 ± 5.7**	196.3 ± 6.2
**LDL (mg/dL)**	98.5 ± 3.7**	103.6 ± 5.6	123.0 ± 5.1**	121.4 ± 5.4
**HDL (mg/dL)**	57.5 ± 2.1*	48.7 ± 2.4*	55.5 ± 2.5	54.6 ± 2.4
**Triglycerides (mg/dL)**	70.9 ± 4.9	95.5 ± 11.1	95.3 ± 12.2	117.2 ± 14.5
**CRP (mg/L)**	1.3 ± 0.2	2.0 ± 0.4	1.8 ± 0.3^	4.6 ± 1.0^

MAP (mean arterial pressure), calculated as MAP = SBP/3 + DBPx2/3 (SBP = systolic blood pressure, DBP = diastolic blood pressure), HR = heart rate, BMI = body mass index, Chol = cholesterol, LDL = low-density lipoprotein cholesterol, HDL = high-density lipoprotein cholesterol, CRP = C-reactive protein.

*P < 0.05, young smokers versus young non-smokers.

^P < 0.05, older smokers versus older non-smokers.

**P < 0.05, young non-smokers versus older non-smokers.

### Magnetic resonance imaging

All MR procedures were performed on a 3 T Siemens TIM Trio imager (Siemens Medical Solutions) using an 8-channel extremity coil for studies at the location of the femoral/popliteal artery and vein (Part I), and a combination of two body matrix and spine coils for PWV of the aorta, iliac and femoral circulation (Part II). All custom-designed pulse sequences were implemented in SequenceTree [25]. Each part of the CMR protocol, including scout scans, lasted approximately 20 mins and the patients were scanned in feet-first supine position.

### CMR assessment of peripheral vascular reactivity

Measures of vascular reactivity were obtained as the post-occlusion change relative to baseline values. Time-resolved blood flow velocity and oxygen saturation (SvO_2_) were measured simultaneously in the femoral artery and vein, respectively (thereby requiring a single cuff occlusion only) with a multi-echo gradient-recalled echo (GRE) sequence described previously [24]. Briefly, lower limb ischemia was induced by applying a blood pressure cuff (SC120D model, Hokanson, Bellevue, WA, USA) to the upper right thigh, i.e. on the adductor longus region. The cuff was inflated to 75 mmHg above the subject’s systolic blood pressure (SBP), but not exceeding 250 mmHg. The cuff paradigm consisted of 2 minutes of baseline, 5 minutes of cuff occlusion, and 6 minutes of recovery. During the baseline (pre-occlusion) period, blood flow velocity and oxygenation were quantified successively. Ten seconds prior to cuff release the pulse sequence was launched to time-resolve arterial blood flow velocity and venous oxygenation simultaneously with temporal resolution of 120 ms and 1.25 s, respectively, for a period of 70 seconds.

**Figure 1:**
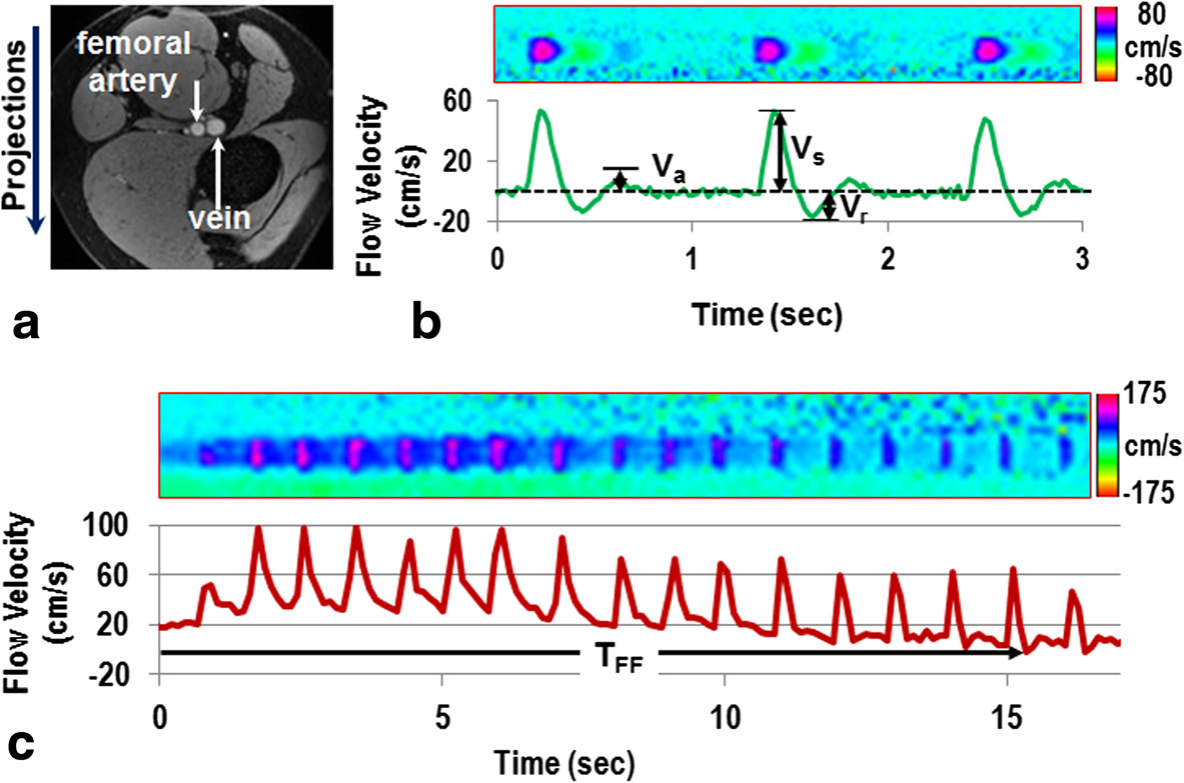
**CMR-based femoral artery blood-flow velocity measurement: a) gray-scale magnitude image showing femoral artery and vein; b) time-resolved velocity (temporal resolution, 20 ms) in the femoral artery at baseline and, c) during hyperemia (temporal resolution, 120 ms).** Projection images in **(b)** display velocity as a function of time for three heart beats, and plots below represent averages across arterial lumen showing normal tri-phasic waveform, with peak systolic velocity, V_s_, peak retrograde velocity, V_r_, and peak antegrade velocity, V_a_, indicated. Dashed line defines the zero velocity. During hypermia after cuff release **(c)** velocity is elevated indicating forward flow only, returning to baseline after approximately 15 s. Horizontal black arrow in **(c)** marks the forward flow duration (i.e. period of hyperemia, T_FF_).

Ungated absolute blood flow velocity was obtained by means of a projection technique [21]. During baseline, the velocity-encoding parameter, referred to as VENC (determining the highest velocity that can be unambiguously measured), was set to 80 cm/s and temporal resolution was 20 ms. During the post-occlusion (hyperemia) period, during which arterial velocity is significantly greater, VENC = 125 cm/s and 175 cm/s were selected, at a temporal resolution of 120 ms (reduction in the temporal resolution results from interleaving velocimetry and oximetry, see below). Sample arterial flow data at baseline and during hyperemia are shown in Figure 1.

**Figure 2:**
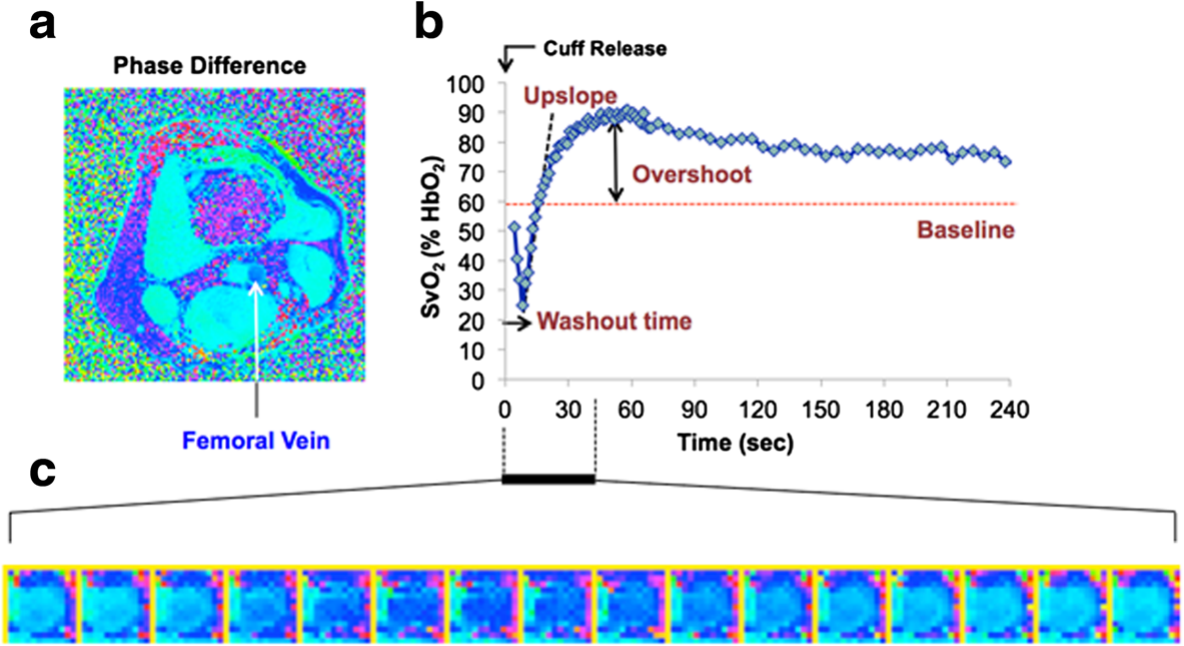
**Quantification of reactive hyperemia with dynamic oximetry. a)** Cross-sectional phase difference image of the thigh, 10 cm below the inferior boundary of the pressure cuff; **b)** post-ischemia (cuff-release at t = 0) femoral vein oxygen saturation (SvO_2_) curve; **c)** zoomed series of phase images showing relative phase of venous blood during period indicated by horizontal black bar. Darker blue represent represents lower saturation levels. Parametrization of the curve in panel **c** yields washout time, time of minimum following cuff release, upslope (slope of the linear portion of the curve following minimum), and overshoot, denoting the maximum of the SvO_2_ curve relative to baseline (preceding cuff contraction, not shown).

The fractional blood oxygenation level (%HbO_2_) in the femoral vein was estimated by field mapping [26], yielding the magnetic susceptibility difference between intravascular blood and surrounding tissue [27]. A representative plot of SvO_2_ versus time, along with phase difference images during hyperemia is shown in Figure 2.

### Measurement of Pulse-Wave Velocity (PWV)

Central and peripheral regional pulse-wave velocities were quantified in the same imaging session following the cuff protocol as described previously [28]. The protocol starts with acquisition of a series of oblique sagittal slices for visualizing the ascending and descending aorta. Multiple axial slices are then acquired below the pulmonary trunk to image both ascending and proximal descending aorta in the same imaging plane. Subsequently, a suitable slice and readout direction are selected for the ungated acquisition of velocity-encoded projections at a temporal resolution of 7.4 ms for about 12 sec to cover 10–12 cardiac cycles. VENC of 180 cm/s was chosen, thereby ensuring accurate mapping of the velocity-time curve with complex difference intensity at the “foot” of the systolic upstroke, which is needed to determine the pulse pressure transit time via “foot-to-foot” method [18].

**Figure 3:**
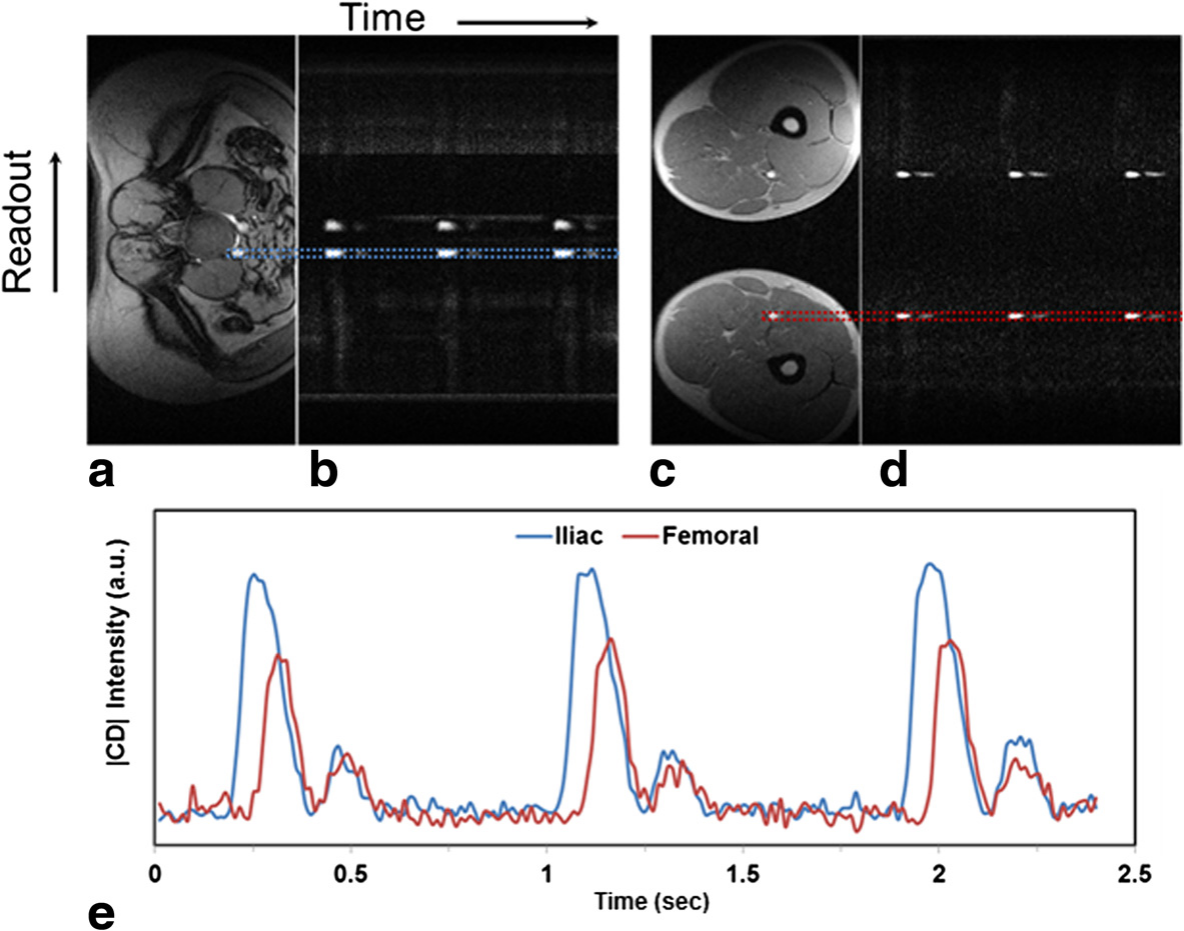
**Measurement of pulse-wave velocity: Reference axial images (superior, a, and inferior, c) and temporally-resolved complex difference intensity (|CD|) images (b, d).** Velocity-encoded projections were acquired simultaneously for quantifying iliofemoral PWV. The systolic peaks are clearly visible on the corresponding velocity images **(b and** **d)**. The time-courses of right iliac and femoral artery |CD| images **(e)** show three cardiac cycles. Note temporal offset in the arrival of the pulse pressure wave in the distal femoral artery with respect to proximal iliac artery. Venous flow velocity is lower by an order of magnitude compared to arterial flow velocity thus they are not visible or interfere with arterial |CD|.

Thoracoabdominal aorta and iliofemoral PWV were obtained analogously except that the velocity-encoded projections were collected simultaneously at two slice locations. For thoracoabdominal aortic PWV measurement velocity-encoded projections were collected at a proximal location of the descending aorta as well as distally in the abdominal aorta (superior to the iliac bifurcation). Finally, for the peripheral iliofemoral PWV the coils were positioned over the abdomen and upper thighs to enable simultaneous acquisition of projections at a slice inferior to the iliac bifurcation and a slice superior to the popliteal artery. The slightly lower temporal resolution of 12 ms is compensated by the greater path length of the pulse pressure wave therefore yielding similar precision at this site. VENC values used were 125 cm/s for thoracoabdominal aorta and 75 cm/s for iliofemoral arteries, respectively, to optimize the complex difference signal intensity. The method is illustrated with sample data in Figure 3.

**Figure 4:**
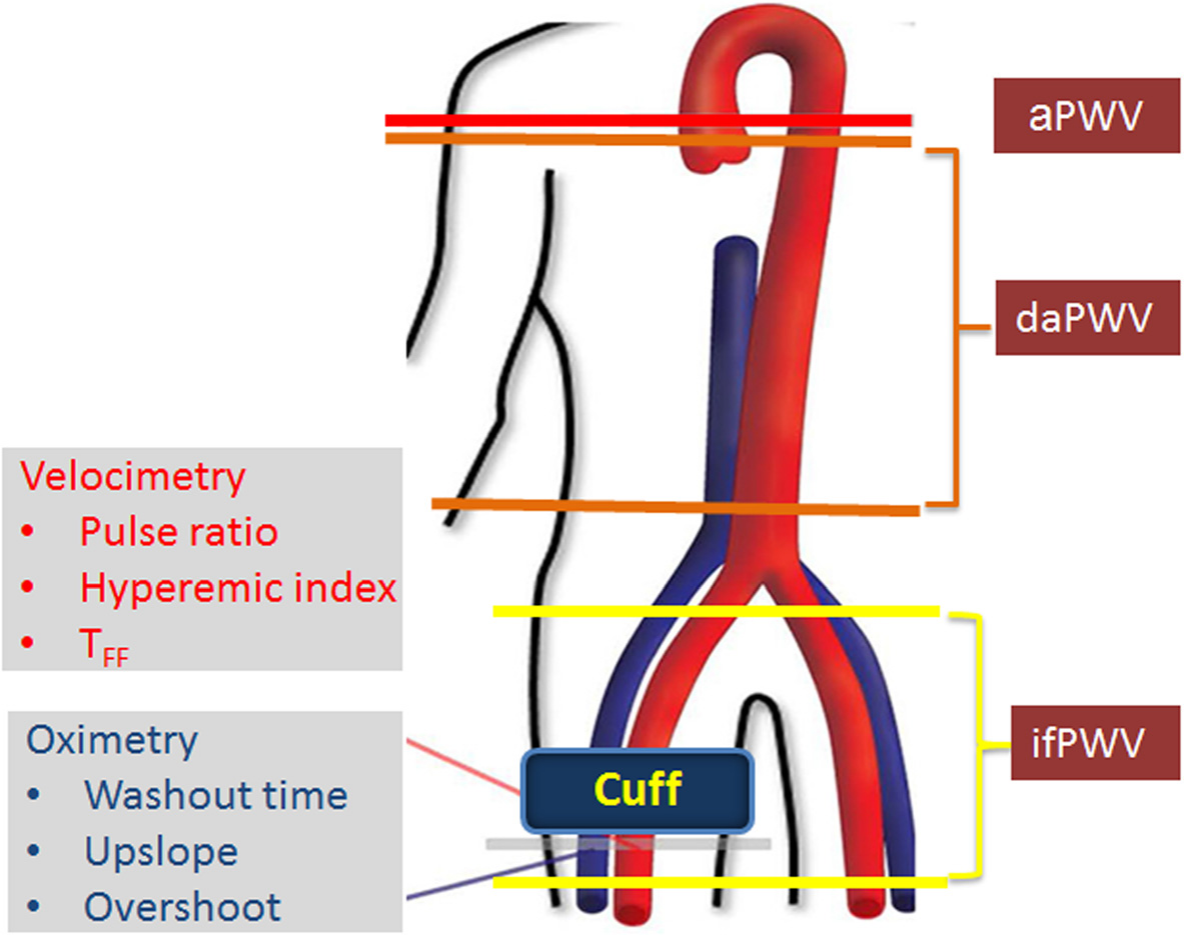
**Schematic of imaging protocol and parameters measured, comprising measurement of vascular reactivity via arterial velocimetry and venous oximetry at rest and hyperemia and measurement of PWV for the three arterial segments indicated: aPWV = ascending aorta PWV, daPWV = descending aorta PWV in the thoracoabdominal segment, if = iliofemoral PWV.**

The integrated CMR protocol and metrics quantified are summarized in Figure 4. The figure graphically illustrates the locations of the measurements described above. All analyses were performed by investigators blinded to patient information.

### CMR data analysis

#### Quantification of time-resolved blood flow velocity in the femoral artery

Baseline (i.e. pre-occlusion) velocities derived from the images (Figure 1a, b) were averaged over multiple heart beats (typically 6–8 beats), yielding peak-systolic velocity (V_s_), peak-retrograde velocity (V_r_), and peak late antegrade velocity (V_a_). From these parameters the pulse ratio (PR) [29], defined as the ratio of systolic and diastolic pulsatility, (V_s_-V_r_)/(V_a_-V_r_), was computed (Figure 1b) instead of the pulsatility index to avoid quantifying average baseline velocity over a cardiac cycle as the latter is predominantly weighted by diastole (low velocity-to-noise ratio) due to the mismatch between VENC (80 cm/s) and blood flow velocity (<5 cm/s). Parameters related to hyperemia included time of forward flow (T_FF_) (Figure 1c), and hyperemic index (HI) defined as the ratio of hyperemic peak flow velocity (V_max_) to (V_a_-V_r_).

#### Quantification of time-resolved SvO_2_ in the femoral vein

First, magnetic field inhomogeneity was corrected to reduce the effects of large-scale static field inhomogeneity [30]. Subsequently, SvO_2_ was computed from the phase difference between the femoral vein and artery, incorporating individual hematocrit level determined from blood sample. The femoral artery, located adjacent to the femoral vein (Figure 2a), was chosen to minimize observer-dependent selection of a reference region [24]. The SvO_2_ time-course curve was parameterized yielding washout time, upslope and overshoot [22] (Figure 2b). The washout time is the time elapsed to observe the oxygen-depleted capillary blood at the imaging slice after cuff deflation at t = 0 s. Upslope and overshoot are defined as the mean resaturation rate during hyperemia and the subsequent maximal above-baseline SvO_2_ level, respectively. Baseline SvO_2_ is the average of four pre-cuff (2 mins baseline period) quantifications.

#### Pulse-wave velocity quantification

The complex difference intensity [31] signals from proximal and distal slices were spatially averaged along the readout direction within the vessel boundary for each time point (temporal resolution = 7.4 ms for aortic arch PWV and 12 ms for thoracoabdominal and iliofemoral PWVs). The time courses from proximal and distal locations were then plotted jointly to determine the propagation time of the wave-front (Figure 3a) [28]. Estimation of the wave-front propagation time illustrated in Figure 3 is equivalent to the foot-to-foot technique commonly utilized in tonometric studies [32]. This measurement was performed for each heart beat and averaged over all cardiac cycles.

For all arterial segments, PWV was calculated as: PWV = L/Δt, where L is the path length of the pressure wave between measurement sites, and Δt is the wave-front propagation time averaged over multiple heartbeats. For the thoracoabdominal aorta and iliofemoral artery segments, path length was determined from the anatomic axial images used for planning purposes. Specifically, the artery’s centroid was manually recorded from the proximal descending aorta to the distal abdominal aorta, corresponding to the measurement locations of the velocity-encoded projections, and path length was computed as the sum of the displacement of the vessel centroid between slices. Similarly for the iliofemoral segment, the path length for the iliofemoral segment was estimated the same way from the iliac bifurcation to the popliteal artery. Finally, total PWV (tPWV) was computed as the sum of path lengths along all three arterial segments divided by the sum of propagation times.

### Ultrasound imaging

High-resolution B-mode ultrasound (US) images along the arterial axis for measuring intimal-medial thickness (IMT) of the common carotid artery, and flow-mediated dilation (FMD) measurements of the brachial artery, were obtained with a broadband high-frequency 9 L4 transducer using a Acuson Sequoia C512 scanner (Siemens Medical Solution USA, Inc. Malvern, PA) by the same, experienced, technician. In general, the US was performed on the day, or within two weeks of the CMR examination. US scans were obtained with subjects at rest in a supine position. The electrocardiogram was continuously monitored and recorded on the ultrasound images. Carotid and brachial artery examinations were performed in the same session.

#### Carotid ultrasound

The segments of the artery imaged included: (1) near wall and far wall of the segment extending from 10 to 20 mm proximal to the tip of the flow divider into the common carotid artery (CCA); (2) near wall and far wall of the carotid bifurcation beginning at the tip of the flow divider and extending 10 mm proximal to the flow divider tip; and (3) near wall and far wall of the proximal 10 mm of the internal carotid artery (ICA). All images were recorded as digital video clips with images triggered on the R-wave.

#### Flow mediated dilation (FMD)

Subject preparation and imaging protocol followed the guidelines outlined by the task force for FMD measurement of the brachial artery [33]. B-mode imaging and spectral Doppler were performed 5–10 cm proximal to the antecubital fossa in the longitudinal plane of the brachial artery. Depth and gain were optimized to identify the lumen-wall interface. B-mode images were recorded as ten-frame video clips with images triggered on R wave. Following the baseline imaging, the pressure cuff, placed above the imaging transducer, was inflated to 200 mm Hg for five minutes. The pressure cuff was released and the spectral Doppler was recorded for the first 10 seconds followed by 2 minutes of imaging. The R-wave triggered B-mode images were recorded for diameter analysis at 1 and 2 minutes post pressure release.

#### Ultrasound image analysis

Carotid images were analyzed for IMT and arterial diameter using automated edge-detection software (Carotid Analyzer for Research v5.0.5, MIA Vascular Research Tools 5, Coralville, IA). Diameter was measured as the distance between media-adventitia margin of the far and near wall. Each measurement was performed in triplicate and bilateral average CCA IMT values were obtained. FMD was determined as the fractional change in brachial artery diameter during hyperemia. Each analysis was repeated three times and the average and standard deviations was reported.

### Statistical analysis

Two-way ANOVA analysis was performed to determine if effect of smoking and age could be separated from one another. If there was no interaction between smoking and age, one-way ANOVA was performed and followed by post-hoc analysis of the parameters between the YNS vs. YS, ONS vs. OS and YNS vs. ONS via unpaired t-tests, with p < 0.05 being considered significant. Nonparametric Wilcoxon tests were performed in situations where parameters were not normally distributed. Further, inter-parameter and inter-modality (CMR versus US metrics) correlations were performed to evaluate associations between parameters derived with different methods and between vascular territories. Associations between conventional cardiovascular risk factor and imaging metrics were also examined using Spearman rank correlation methods. All analyses were performed in JMP (JMP®, Version 11, SAS Institute Inc., Cary, NC).

## Results

Two-way ANOVA indicated that there was no interaction between smoking and age (P = 0.40 -0.89) for all parameters evaluated, which allowed combining smokers in the young and older sub-groups and compare with all nonsmokers. Additional comparisons of interest reported in Table 2 are between old and young nonsmokers (age effect) and smokers and nonsmokers within each of the two age groups (smoking effect).

### Arterial blood flow at baseline and hyperemia

**Figure 5:**
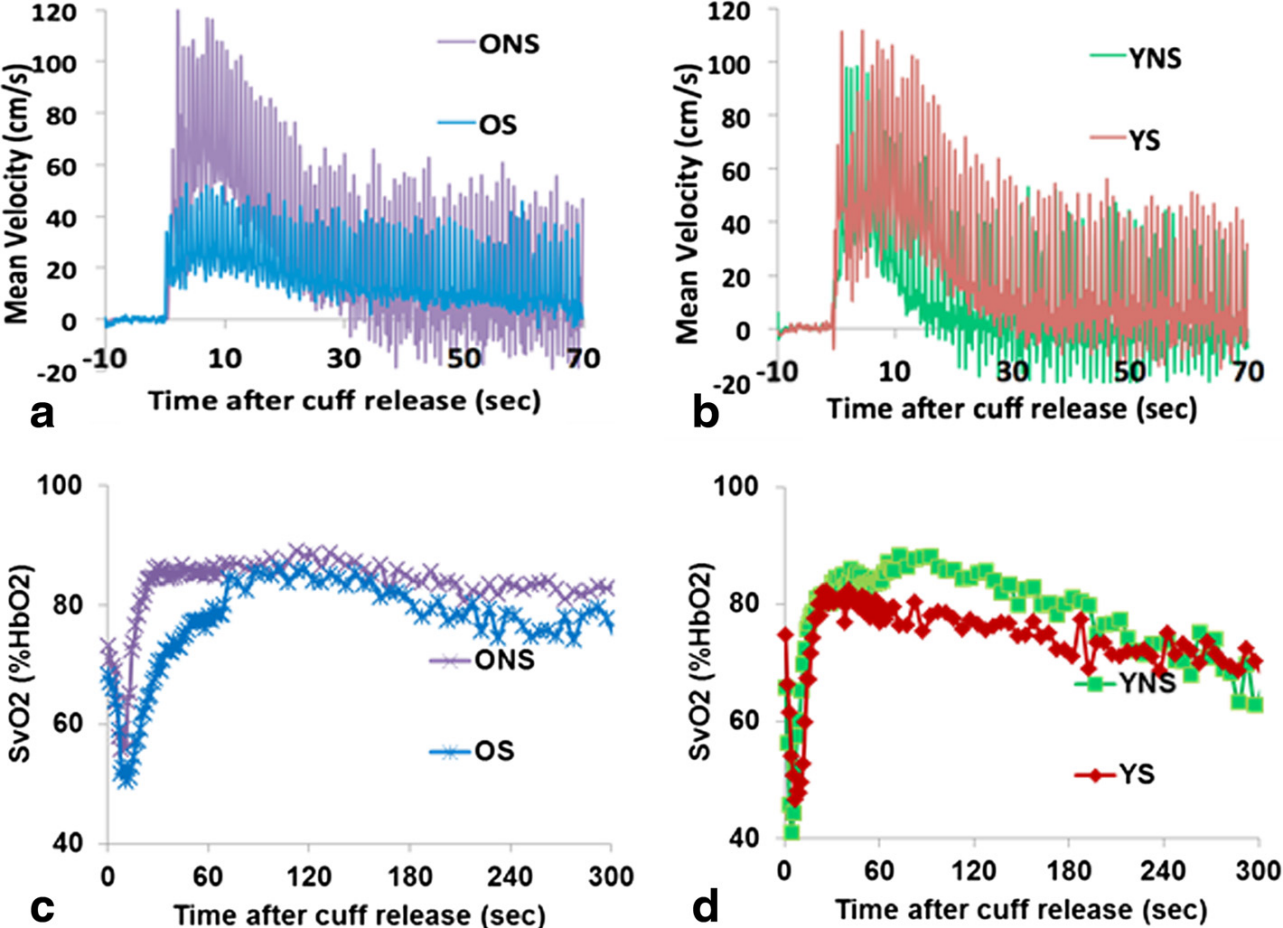
**Post-cuff occlusion time course of blood flow velocity and SvO2 in femoral artery and vein, respectively. a, b)** Femoral artery velocity during hyperemia, comparing ONS to OS **(a)**, and YNS to YS **(b)** in representative subjects. Note greater initial amplitude and shorter forward flow (T_FF_) in nonsmokers; **c, d)** corresponding comparisons for femoral vein SvO_2_ time-course.

At baseline, the blood flow displays the typical tri-phasic pattern during the cardiac cycle (systolic, retrograde and late antegrade) (Figure 1b). However, with advancing age the velocity wave is increasingly damped, as is evident from the results in Table 2 showing that older subjects had significantly greater pulse ratio (i.e. reduced diastolic pulsatility, V_a_-V_r_) than their younger peers (3.51 ± 0.08 versus 3.09 ± 0.08, P < 0.001). During hyperemia, blood flow velocity is elevated, proceeding to a mono-phasic pattern, eventually returning to baseline in 10–40 s (Figure 1c). Smokers overall had lower hyperemic index than non-smokers (1.01 ± 0.01 versus 1.25 ± 0.01, P < 0.01). None of the other arterial flow measures differed significantly among groups. Figure 5a,b show representative time-courses of blood flow velocity in the femoral artery during hyperemia for each of the four groups.

**Table 2:** **Means and standard errors of CMR parameters derived from dynamic venous oximetry, arterial velocimetry and pulse-wave velocity measurements, as well as inter-group differences**

**Metrics**	**Y**	**O**	**NS**	**S**	**YNS**	**YS**	**ONS**	**OS**	**Differences (%)**				
									**O-Y**	**S-NS**	**ONS-YNS**	**YS-YNS**	**OS-ONS**
**Dynamic oximetry**													
**Overshoot (%HbO**_**2**_ **)**	18.5±0.97	13.8±0.99	17.0±0.97	15.2±1.05	19.9±1.50	17.1±1.28	14.0±1.35	13.4±1.31	–25.6***	–10.7	–29.4**	–13.7	–4.4
**Upslope (%HbO**_**2**_ **/s)**	3.34±0.17	2.43±0.17	3.33±0.16	2.39±0.18	3.74±0.21	2.86±0.25	2.89±0.24	1.94±0.22	–27.2***	–28.4***	–22.7**	–23.6**	–33.0**
**Washout time (s)**	9.53±0.38	10.6±0.39	9.37±0.37	10.8±0.40	9.00±0.31	10.2±0.51	9.77±0.44	11.4±0.84	11.1	15.3**	8.6	13.5*	16.3
**Dynamic velocimetry**													
**Pulse ratio**	3.09±0.08	3.51±0.08	3.32±0.08	3.28±0.08	3.09±0.13	3.08±0.10	3.55±0.10	3.47±0.11	13.5***	−1.2	14.9**	−0.5	−2.5
**Hyperemic index**	1.16±0.01	1.12±0.01	1.25±0.01	1.01±0.01	1.24±0.07	1.06±0.08	1.27±0.10	0.97±0.08	–3.9	–19.5**	2.1	–14.9	–23.4*
**Forward flow time-T**_**FF**_ **(s)**	33.2±1.66	30.8±1.62	33.1±1.6	30.2±1.68	34.7±2.06	30.3±2.22	31.5±2.35	30.2±2.57	–7.2	–8.7	–9.3	–12.8	–3.9
**Pulse-wave velocity (m/s)**													
**Aortic arch**	8.16±0.25	9.37±0.24	8.46±0.25	9.16±0.26	7.94±0.27	8.46±0.35	8.98±0.32	9.77±0.44	14.8***	8.2	13.1*	6.5	8.8
**Thoraco-abdominal**	4.87±0.25	7.43±0.24	6.12±0.28	6.31±0.29	4.69±0.15	5.08±0.17	7.53±0.53	7.33±0.36	52.5***	3.0	60.7***	8.5	–2.6
**Iliofemoral**	6.78±0.21	8.08±0.21	7.31±0.22	7.57±0.23	6.72±0.24	6.79±0.23	7.91±0.26	8.26±0.43	19.2***	3.6	17.7**	1.0	4.5
**Total**	5.93±0.13	7.54±0.13	6.65±0.15	6.88±0.16	5.80±0.10	6.06±0.13	7.48±0.20	7.60±0.26	27.1***	3.5	29.0***	4.4	1.6

Y = young subjects, O = older subjects, NS = non-smoking, S = smoking. Relative differences: ***P ≤ 0.001, **P ≤ 0.01, *P < 0.05.

### Venous oxygen saturation at baseline and hyperemia

**Figure 6:**
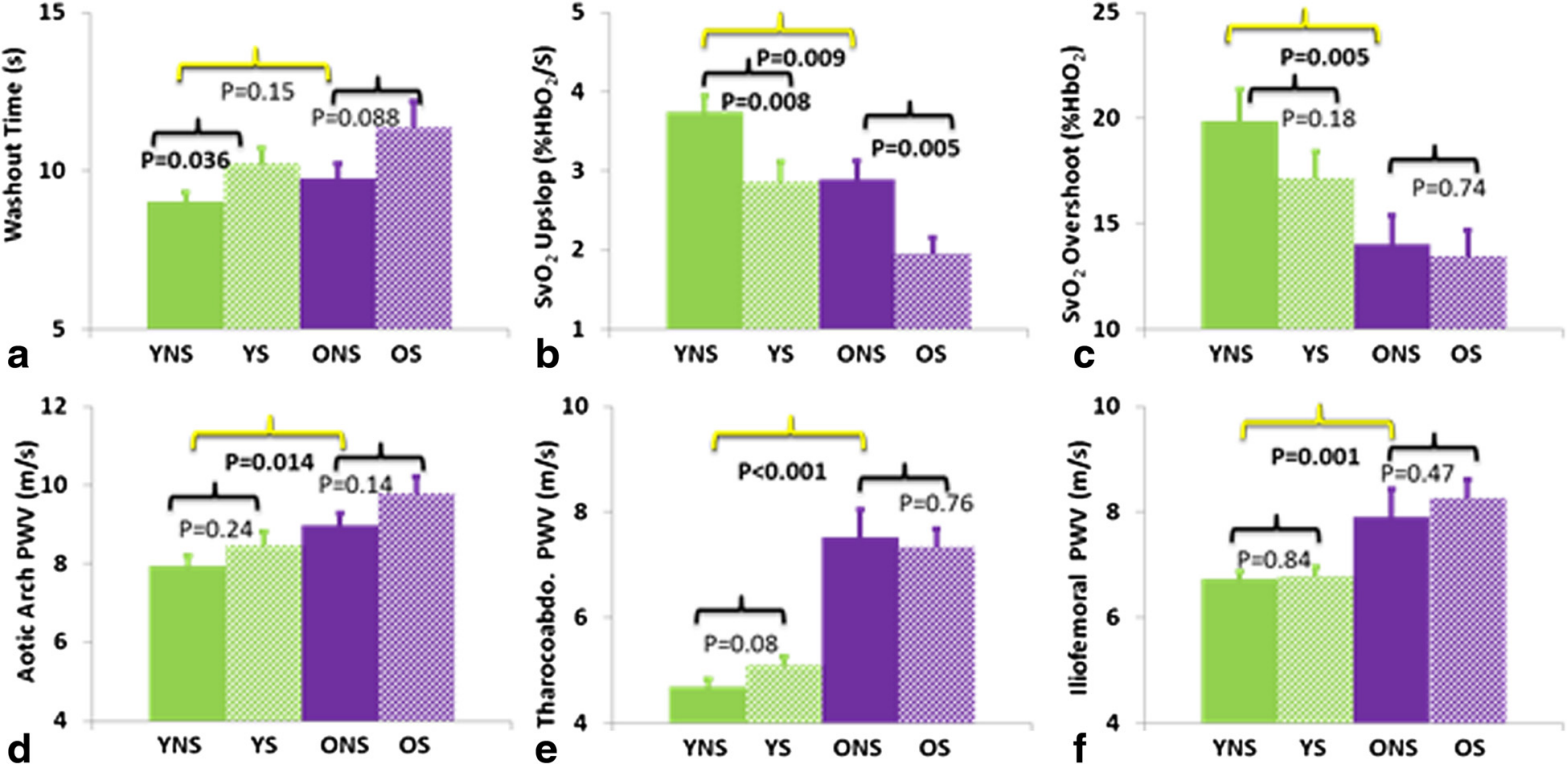
**Summary of most relevant inter-group comparisons: a-c) dynamic venous oximetry; d-f) PWV.** Data suggest prolonged washout time in smokers **(a)**, reduced rate of re-saturation (upslope) in both smokers and older subjects **(b)**, and similarly, lower overshoot **(c)**. Age-related increased arterial stiffness is evident for all three arterial segments **(d-f)**. Significant p-values (P < 0.05) were highlighted in bold.

Figure 5c,d display venous oxygen saturation curves during hyperemia for representative subjects comparing smokers and nonsmokers. Collectively, older subjects had significantly lower SvO_2_ overshoot compared to their younger peers (13.8 ± 0.99 vs. 18.5 ± 0.97%HbO_2_, P < 0.001), as well as lower SvO_2_ upslope (2.43 ± 0.17 vs. 3.34 ± 0.17%HbO_2_/s, P < 0.001). Old non-smokers presented with lower overshoot (14.0 ± 1.35 vs. 19.9 ± 1.50%HbO_2_, P < 0.001) and lower SvO_2_ upslope ratio (2.89 ± 0.24 vs. 3.74 ± 0.21%HbO_2_/s, P < 0.01) than young non-smokers (Table 2). Smokers (young and old combined), had lower SvO_2_ upslope compared to their non-smoking peers (2.39 ± 0.18 vs. 3.33 ± 0.16%HbO_2_/s, P < 0.01). Comparing only smokers to nonsmokers within an age group, young smokers had lower SvO_2_ upslope (2.86 ± 0.25 vs. 3.74 ± 0.21%HbO_2_/s, P < 0.01), and longer washout time (10.2 ± 0.51 vs. 9.00 ± 0.31 s, P < 0.05). The data are commensurate with the notion of age- and smoking-related impaired microvascular reactivity (Figure 6).

### Pulse wave velocity

The pooled data in Table 2 comparing the two age groups (old smokers and nonsmokers versus their younger peers) indicate PWV for all segments as well as tPWV to be highly significantly greater in the older subject group (P < 0.001), and primarily in the non-smoking groups (P < 0.05). However, there was no significant difference between smokers and nonsmokers even though for some segments the differences approached significance, as for example for the thoracoabdominal aorta (P = 0.08). Subgroup analysis comparing smokers to nonsmokers within each age group did not reveal significant differences. Nevertheless, the data are suggestive of smokers to have greater PWV than nonsmokers for all vascular segments evaluated (Figure 6).

### Ultrasound measures

Ultrasound data are compiled in Table 3. Both right and left common carotid artery (CCA) IMT was greater in the older than in the young subject group (0.66 ± 0.01 vs. 0.49 ± 0.01 mm, P < 0.001, and 0.70 ± 0.02 vs. 0.49 ± 0.02 mm, P < 0.001, respectively). Importantly, left CCA IMT was also greater in smokers compared to non-smokers (0.63 ± 0.02 vs. 0.57 ± 0.02 mm, P < 0.05), especially in young smokers compared to their nonsmoking peers (0.53 ± 0.02 vs. 0.46 ± 0.01 mm, P < 0.05). However, the difference in the right CCA IMT between smokers and non-smokers was not significant. There were no significant inter-group differences in FMD even though there was a general trend of reduced FMD in smokers and older subjects (P = 0.15 - 0.89).

**Table 3:** **Means and standard errors for ultrasound derived measures (IMT and FMD), as well as inter-group differences**

**Metrics**	**Y**	**O**	**NS**	**S**	**YNS**	**YS**	**ONS**	**OS**	**Differences (%)**				
									**O-Y**	**S-NS**	**ONS-YNS**	**YS-YNS**	**OS-ONS**
**Right CCA IMT (mm)**	0.49 ± 0.01	0.66 ± 0.01	0.56 ± 0.02	0.60 ± 0.02	0.46 ± 0.01	0.52 ± 0.02	0.66 ± 0.02	0.68 ± 0.03	34.8***	6.8	41.9***	12.8	3.2
**Left CCA IMT (mm**	0.49 ± 0.02	0.70 ± 0.02	0.57 ± 0.02	0.63 ± 0.02	0.46 ± 0.01	0.53 ± 0.02	0.68 ± 0.02	0.72 ± 0.03	41.3***	9.9*	49.3***	16.6*	5.8
**1-min FMD (%)**	7.83 ± 0.69	7.04 ± 0.61	7.79 ± 0.63	6.93 ± 0.67	7.96 ± 0.96	7.66 ± 0.92	7.65 ± 0.96	6.38 ± 0.82	−10.1	−11.1	−3.9	−3.8	−16.6
**2-min FMD (%)**	6.61 ± 0.7	6.25 ± 0.64	6.85 ± 0.65	5.94 ± 0.68	6.99 ± 0.86	6.11 ± 0.92	6.71 ± 0.93	5.80 ± 1.03	−5.5	−13.3	−4.0	−12.6	−13.6

Y = young subjects, O = older subjects, NS = non-smoking, S = smoking.

Relative differences: ***P ≤ 0.001, *P < 0.05.

### Correlations of CMR parameters with other metrics

Inter-parameter correlations are listed in Table 4. While correlations between parameters derived within the same anatomic territory and representing similar properties (e.g., parameters characterizing the kinetics of oxygen re-saturation during hyperemia, or arterial flow velocity at baseline and hyperemia) are unsurprising, of interest are associations between measures representative of different vascular territories or between oximetric and arterial flow measures. For example descending aortic PWV was negatively correlated with SvO_2_ upslope (r = −0.31, P < 0.001). The relationship between increased stiffness of central artery and reduced vascular reactivity further supports the hypothesis that increased PWV leads to increased transmission of deleterious pulse pressure to the peripheral bed [34].

**Table 4:** **Spearman correlation coefficients for inter-parameter correlations of CMR measures involving the entire cohort (N = 169)**

**Correlation**	**Oximetry**						**PWV**		
	**OS**	**Upslope**	**Washout**	**PR**	**HI**	**T**_**FF**_	**aPWV**	**daPWV**	**ifPWV**
**Washout**	-								
**Upslope**	**0.45**	-							
**OS**	−0.14	**−0.42**	-						
**PR**	**−0.34**	−0.17	0.11	-					
**HI**	**0.17**	**0.23**	−0.10	0.04	-				
**T**_**FF**_	−0.02	−0.01	−0.04	**0.19**	**0.31**	-			
**aPWV**	**−0.22**	−0.12	0.05	**0.22**	0.04	0.01	-		
**daPWV**	**−0.19**	**−0.31**	**0.24**	**0.22**	0.00	−0.07	**0.23**	-	
**ifPWV**	−0.12	−0.14	0.09	**0.18**	0.10	0.09	**0.35**	**0.29**	-

aPWV = aortic arch PWV, daPWV = thoracoabdominal aortic PWV, and ifPWV = iliofemoral arterial PWV.

Significant correlations (P < 0.05, corresponding to |r| > 0.15) are bolded.

**Figure 7:**
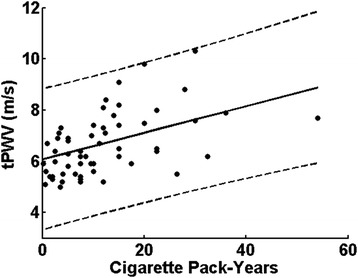
**Significant correlation between smoking severity (cigarette pack-years) and total PWV (tPWV) along three arterial segments in smokers was found (r = 0.52, P < 0.001).** Dotted line denotes 95% confidence interval of the linear fitting of cigarette pack-years to tPWV.

Significant associations were also seen between imaging measures and cardiovascular risk factors. All PWVs correlated with SBP (r = 0.27-0.42, P < 0.001). In non-smokers, the BMI and C-reactive protein levels correlated with microvascular CMR parameters, including forward flow time T_FF_, HI, and PR as well as SvO_2_ overshoot, upslope and washout time (r = 0.16 – 0.97, P < 0.05). Further, total PWV, calculated as a total path length divided by the total transit time over the three arterial segments, correlated with cumulative cigarette smoke exposure expressed in terms of pack years in smokers (r = 0.52, P < 0.001, Figure 7). Lastly, carotid IMT in young smokers correlated with PWV at all three locations (r = 0.21 – 0.45, all P < 0.01), similarly between IMT and microvascular CMR parameters including PR and HI, SvO_2_ overshoot, upslope and washout time (r = 0.16-0.53, P < 0.05). No other significant correlations were found between CMR metrics and either laboratory findings or ultrasound FMD results.

## Discussion and conclusions

The focus of the present study was to evaluate the effects of aging and smoking on surrogate markers of endothelial function in the form of new quantitative CMR measures obtained in the peripheral and central vasculature. The approach chosen is unique in that it allows quantification of parameters of microvascular as well as macrovascular functional and mechanical properties (response to hyperemia, arterial wave-form analysis and arterial compliance) as part of a single, integrated examination using novel CMR methods and comparing results to conventional ultrasound measures. The study population was chosen so as to allow detection of early, preclinical manifestations of noninvasively measurable parameters in subjects without overt cardiovascular disease. One important and unexpected outcome of the study is the finding that for all functional parameters evaluated the effects of smoking and age were independent of one another.

Vascular reactivity has predominantly been studied by flow-mediated dilation of the brachial artery via B-mode ultrasound [33], which has provided insights into abnormal endothelial function in subjects with cardiovascular risk factors, and in relationship to age and smoking [35-37]. Here, the main target toward assessment of microvascular reactivity and endothelial function was the femoro-popliteal circulation, given that atherosclerotic disease is far more prevalent in the lower than in upper extremities. New CMR methods recently introduced by some of the present authors allow simultaneous evaluation of both arterial as well as venous response to cuff-induced ischemia [21]. The two approaches are complementary, mapping the temporal evolution of arterial velocity via high temporal-resolution, projection-based, phase-contrast imaging [28], and SvO_2_ via field mapping, which is measured dynamically during hyperemia in the femoral vein [21] (see Figure 2).

For the latter method, washout time, the period between restoration of flow after cuff-occlusion, and the minimum of the SvO_2_-time curve, was significantly prolonged in the young smoking group. Similarly, the rate of recovery, termed upslope, expressed in terms of the rate of change, Δ(SvO_2_)/Δt, was lower in the older relative to that in the younger subject group. Further, overshoot, the transient increase above baseline, of the SvO_2_-time curve, was reduced in the older compared to the younger study subjects. Importantly, in both young and older study groups, smokers exhibited similar impairments of endothelial function in terms of the three oximetric parameters (see Figure 6a-c). Complementing the hyperemic oximetry data was the hyperemic index (HI) following cuff deflation, which in the older subject group was significantly lower in smokers relative to nonsmokers.

Our findings are commensurate with the notion that both age and smoking promote impaired, nitric oxide dependent, microvascular reactivity [38]. There exist no data in the literature other than our own in patients with peripheral arterial disease, in which the dynamic venous oximetric parameters proved to be strong differentiators of subjects with peripheral arterial disease, from controls [21,22]. Brachial artery FMD, measured in the present study for comparison, has long been regarded as a biomarker for NO-dependent endothelial function (see, for example, ref. [39] and thus provides a means for comparison. In a small quantitative CMR study comparing young smokers to matched nonsmokers, Wiesmann et al. had found reduced brachial artery FMD measured from the change in cross-sectional area of the brachial artery [20]. These findings parallel those by Celermajer et al. [35], who found in a cohort of 200 subjects divided into controls, current and former smokers, all normotensive without a history of premature vascular disease, that brachial artery FMD, measured by B-mode US, to be reduced in smokers. Our reference, brachial-artery FMD data obtained via B-mode ultrasound, suggest lower FMD in smokers within an age group, as well as in older compared to younger nonsmokers, albeit without reaching significance, which might be due to the relatively small group sizes. On the other hand, the baseline femoral artery velocity waveform, characterized in terms of the pulse ratio (a measure of the extent of damping of the pulsatility during diastole), was significantly higher in the older relative to the younger nonsmoking subject group, indicative of the vessel’s impaired viscoelastic properties.

The second element of our CMR protocol centered on the evaluation of pulse-wave velocity along two successive aortic segments (aortic arch, thoracoabdominal aorta), involving image-based measurement of the path length. We also assessed iliofemoral PWV, which has not previously been reported. The data are of interest given that conventionally-derived, carotid to femoral PWV, has been shown to be positively correlated with age [40] while the effect of smoking yielded less conclusive results (see, for instance, Doonan et al. [19] for a meta analysis). Our data were strongly associated with age, indicating older non-smokers to have elevated PWV in all three segments relative to their younger peers (Figure 6 and Table 2). However, the thoracoabdominal segment demonstrated the largest age effect where group means of older nonsmokers were 60% greater than those in the younger nonsmoking subject group, in agreement with recently reported CMR-based regional measurements [41]. Further, our results indicate positive correlations with SBP and PWV for all three segments as well as with total PWV. The data in Figure 6d-f suggest that within an age group smokers had increased PWV relative to nonsmokers, but these relationships did not reach significance. However, when plotted against smoking severity, total PWV was found to increase with the number of pack-years (Figure 7).

Recently, Koivistoinen et al. [42] reported an inverse relationship between carotid distensibility and IMT, suggesting that elevated PWV may be governed by similar factors that determine preclinical increases in arterial wall thickness. Even though our pulse-wave velocities were not measured at the same anatomic location, we found the values in the three segments to be positively correlated with carotid IMT, indicative of a systemic effect. Our IMT data were strongly associated with both age and smoking, showing mean values on both sides to be significantly greater than those in the younger subject group. Finally, IMT in young smokers was elevated relative to that of their nonsmoking peers, in line with early data, which provided evidence for smoking to contribute independently to increased IMT [43].

Both, the methods used, and study design, have limitations. The analysis of the raw data, in particular the time-course measures, is involved and operator-intensive, and will need to be streamlined for future large-scale studies. However, we enrolled only subjects with BMI < 30, which excludes a substantial fraction of the general population. As far as the study itself is concerned, there may be confounds masking the effect of smoking, such as lifestyle factors (e.g. exercise), and the lifetime exposure to smoking, which need to be captured in future follow-up studies. The demographics in Table 1 may suggest race as a possible confound. Since, as previously stated, two-way ANOVA did not show a statistically significant interaction between age and smoking, regression with and without race as a variable was performed to determine if the covariate changed the effects of age and smoking. The results of the analysis showed no statistically significant effect in any of the MR parameters. Thus, the slight imbalance in racial distribution should not affect the work’s overall conclusions.

Unique to the approach presented in the present work is CMR’s ability to quantify a spectrum of biomarkers of vascular health, both at the micro- and macrovascular level, as part of a single examination. The work is the first to examine the effects of cigarette smoking and aging on new measures of vascular reactivity and endothelial dysfunction in subjects without symptomatic cardiovascular disease. Unlike ultrasound, where inter-operator errors (for example from transducer placement) adversely affect repeatability, CMR is more stable as the choice of the measurement location is entirely image-guided.

In summary, while these early data are promising, corroborative studies in larger cohorts will be needed, along with rigorous comparison with more established methods. Of particular interest will be to ascertain whether the methods are able to quantify longitudinal changes such as those resulting as a consequence of aging or smoking cessation.
